# How to assess and compare inter-rater reliability, agreement and correlation of ratings: an exemplary analysis of mother-father and parent-teacher expressive vocabulary rating pairs

**DOI:** 10.3389/fpsyg.2014.00509

**Published:** 2014-06-04

**Authors:** Margarita Stolarova, Corinna Wolf, Tanja Rinker, Aenne Brielmann

**Affiliations:** ^1^Department of Psychology, University of KonstanzKonstanz, Germany; ^2^Zukunftskolleg, University of KonstanzKonstanz, Germany; ^3^Department of Society and Economics, Rhine-Waal University of Applied SciencesKleve, Germany; ^4^Department of Linguistics, University of KonstanzKonstanz, Germany

**Keywords:** inter-rater agreement, inter-rater reliability, correlation analysis, expressive vocabulary, parent questionnaire, language assessment, parent–teacher ratings, concordance of ratings

## Abstract

This report has two main purposes. First, we combine well-known analytical approaches to conduct a comprehensive assessment of agreement and correlation of rating-pairs and to dis-entangle these often confused concepts, providing a best-practice example on concrete data and a tutorial for future reference. Second, we explore whether a screening questionnaire developed for use with parents can be reliably employed with daycare teachers when assessing early expressive vocabulary. A total of 53 vocabulary rating pairs (34 parent–teacher and 19 mother–father pairs) collected for two-year-old children (12 bilingual) are evaluated. First, inter-rater reliability both within and across subgroups is assessed using the intra-class correlation coefficient (ICC). Next, based on this analysis of reliability and on the test-retest reliability of the employed tool, inter-rater agreement is analyzed, magnitude and direction of rating differences are considered. Finally, Pearson correlation coefficients of standardized vocabulary scores are calculated and compared across subgroups. The results underline the necessity to distinguish between reliability measures, agreement and correlation. They also demonstrate the impact of the employed reliability on agreement evaluations. This study provides evidence that parent–teacher ratings of children's early vocabulary can achieve agreement and correlation comparable to those of mother–father ratings on the assessed vocabulary scale. Bilingualism of the evaluated child decreased the likelihood of raters' agreement. We conclude that future reports of agreement, correlation and reliability of ratings will benefit from better definition of terms and stricter methodological approaches. The methodological tutorial provided here holds the potential to increase comparability across empirical reports and can help improve research practices and knowledge transfer to educational and therapeutic settings.

## 1. Introduction

When it comes to the usability of screening tools both validity and reliability of an instrument are important quality indicators. They are needed to estimate the usefulness of assessments in therapeutic, educational and research contexts and are therefore highly relevant in a variety of scientific disciplines, such as psychology, education, medicine, linguistics and others that often rely on ratings to evaluate behaviors, symptoms or abilities. Validity is defined as—the degree to which evidence and theory support the interpretations of scores entailed by proposed uses of tests—(American Educational Research Association et al., 1999). In a way, validity of an assessment instrument mirrors its ability to capture, what it intends to measure. Reliability estimates describe the precision of an instrument, they refer to its capacity to produce constant, similar results. There are different possibilities to measure reliability, e.g., across raters that evaluate the same participant (inter-rater reliability) or across different points in time (test-retest reliability, for a comprehensive discussion on validity and reliability see for example, Borsboom et al., 2004). Reliability estimates for example of children's language capacities are often restricted to linear correlations and lack precise understanding of methodological approaches, which can lead to significant limitations regarding the interpretability and comparability of the reported results. This article therefore aims to provide a methodological tutorial for assessing inter-rater reliability, agreement and correlation of expressive vocabulary ratings. By applying the proposed strategy to a concrete research question, i.e., whether a screening questionnaire developed for use with parents can be employed also with daycare teachers, we are able to show the potential impact of using different measures of reliability, agreement and correlation on the interpretation of concrete empirical results. The proposed approach can potentially benefit the analyses of ratings regarding a variety of abilities and behaviors across different disciplines.

Extensive research has provided evidence for the validity of language screening tools such as the German vocabulary questionnaire ELAN (Eltern Antworten, Bockmann and Kiese-Himmel, 2006) used in this study and similar instruments (e.g., the MacArthur-Bates CDI scales, Fenson, 1993, 2007) not only with regard to parental, but also to teacher evaluations Marchman and (Martinez-Sussmann, 2002; Norbury et al., 2004; Bockmann, 2008; Vagh et al., 2009). Most of the validity studies correlate vocabulary ratings with objective lexical measures, such as for example the Peabody Picture Vocabulary Test (Dunn and Dunn, 2007) and find strong associations between the scores children achieve in an objective test situation and the vocabulary ratings provided by different caregivers, e.g., mothers, fathers, or teachers 9Janus, 2001; Norbury et al., 2004; Bishop et al., 2006; Massa et al., 2008; Koch et al., 2011).

In contrast to validity of parental and teacher ratings regarding expressive vocabulary, their reliability has not been sufficiently substantiated, specifically with regard to caregivers other than parents. Since a significant number of young children are experiencing regular care outside their families, the ability of different caregivers to provide a reliable assessment of behavior, performance or ability level, using established tools, is relevant with regard to screening and monitoring a variety of developmental characteristics (e.g., Gilmore and Vance, 2007). The few studies examining (inter-rater) reliability regarding expressive vocabulary frequently rely solely or predominantly on linear correlations between the raw scores provided by different raters (e.g., de Houwer et al., 2005; Vagh et al., 2009.) Moderate correlations between two parental ratings or between a parent and a teacher rating are reported, varying between *r* = 0.30 and *r* = 0.60. These correlations have been shown to be similar for parent–teacher and father–mother rating-pairs (Janus, 2001; Norbury et al., 2004; Bishop et al., 2006; Massa et al., 2008; Gudmundsson and Gretarsson, 2009; Koch et al., 2011).

While the employed correlation analyses (mostly Pearson correlations) provide information about the strength of the relation between two groups of values, they do not capture the agreement between raters at all (Bland and Altman, 2003; Kottner et al., 2011). Nonetheless, claims about inter-rater agreement are frequently inferred from correlation analyses (see for example, Bishop and Baird, 2001; Janus, 2001; Van Noord and Prevatt, 2002; Norbury et al., 2004; Bishop et al., 2006; Massa et al., 2008; Gudmundsson and Gretarsson, 2009.) The flaw of such conclusions is easily revealed: A perfect linear correlation can be achieved if one rater group systematically differs (by a nearly consistent amount) from another, even though not one single absolute agreement exists. In contrast, agreement is only reached, when points lie on the line (or within an area) of equality of both ratings (Bland and Altman, 1986; Liao et al., 2010). Thus, analyses relying solely on correlations do not provide a measure of inter-rater agreement and are not sufficient for a concise assessment of inter-rater reliability either. As pointed out by Stemler (2004), reliability is not a single, unitary concept and it cannot be captured by correlations alone. To show how the three concepts inter-rater reliability expressed here as intra-class correlation coefficients (ICC, see Liao et al., 2010; Kottner et al., 2011), agreement (sometimes also termed consensus, see for example, Stemler, 2004), and correlation (here: Pearson correlations) complement each other in the assessment of ratings' concordance is one main intention of this report.

Conclusions drawn from ratings provided by different raters (e.g., parents and teacher) or at different points of time (e.g., before and after an intervention) are highly relevant for many disciplines in which abilities, behaviors and symptoms are frequently evaluated and compared. In order to capture the degree of agreement between raters, as well as the relation between ratings, it is important to consider three different aspects: (1) inter-rater reliability assessing to what extent the used measure is able to differentiate between participants with different ability levels, when evaluations are provided by different raters. Measures of inter-rater-reliability can also serve to determine the least amount of divergence between two scores necessary to establish a reliable difference. (2) Inter-rater agreement, including proportion of absolute agreement, where applicable also magnitude and direction of differences. (3) Strength of association between ratings, measured by linear correlations. Detailed explanations of these approaches are provided for example by Kottner and colleagues in their “Guidelines for Reporting Reliability and Agreement Studies” (Kottner et al., 2011). Authors from the fields of education (e.g., Brown et al., 2004; Stemler, 2004) and behavioral psychology (Mitchell, 1979) have also emphasized the necessity to distinguish clearly between the different aspects contributing to the assessment of ratings' concordance and reliability. Precise definition and distinction of concepts potentially prevents misleading interpretations of data. As the different but complementary concepts of agreement, correlation and inter-rater reliability are often mixed up and these terms are used interchangeably (see e.g., Van Noord and Prevatt, 2002; Massa et al., 2008), below we briefly present their definitions and methodological backgrounds, while also linking each of them to the content related questions addressed in the present report.

The term agreement (or consensus) refers to the degree to which ratings are identical (for detailed overviews see, de Vet et al., 2006; Shoukri, 2010) often described using the proportion of identical to diverging rating pairs (Kottner et al., 2011). In order to state, however, whether two ratings differ statistically from each other, psychometric aspects of the employed tool, such as reliability (e.g., test-retest reliability or intra-class correlations as a measure of inter-rater reliability), must be taken into consideration. General characteristics of the rating scale, for example the presence or absence of valid scoring categories (Jonsson and Svingby, 2007) and the number of individual items (and thus decisions) comprising a score, will influence directly the likelihood of absolute agreement. For example, the more items a scale comprising a raw-score has, the less likely it is to reach absolute agreement of scores. Therefore, two raw scores or two standardized values (such as *T*-scores) diverging in absolute numbers are not necessarily statistically different from each other. An (absolute) difference can be too small to reflect a systematic divergence in relation to the distribution of scores. Thus, the size of non-systematic errors has to be taken into account prior to making judgments on proportions of agreement. Unfortunately, many studies attempting to assess inter-rater agreement completely disregard the distinction between absolute differences and statistically reliable differences and do not use standardized values (e.g., Bishop and Baird, 2001; Bishop et al., 2006; Gudmundsson and Gretarsson, 2009). In the field of language acquisition for example the direct comparison of raw-scores still seems to be the norm, rather than the exception, despite the lengthy item lists comprising vocabulary assessment instruments (e.g., Marchman and Martinez-Sussmann, 2002; Norbury et al., 2004).

Before assessing absolute agreement, it is thus necessary to determine the minimum divergence classifying two ratings as statistically (and thus reliably) different. One way to establish reliable difference is to calculate the so called “Reliable Change Index” (RCI, e.g., Zahra and Hedge, 2010) an approach intended to define significantly changed or diverging values. If the RCI is significant, a 95% probability that the two values differ from each other can be assumed. Critically, the RCI is a function of the employed instrument's reliability. There are several reliability measures appropriate for calculating the RCI, among them test-retest or inter-rater reliability. However, different reliability measures are likely to yield different results, depending mostly on the characteristics of the population samples they are derived from. For a standardized instrument such as the vocabulary checklist ELAN (Bockmann and Kiese-Himmel, 2006), reliability assessments derived from the standardization sample (e.g., the test-retest reliability according to the instrument's manual) provide a conservative estimate of its reliability. Reliability for calculating the RCI can also be estimated for a concrete study sample, which is usually smaller and often less representative than the standardization sample. This second approach is thus likely to provide a less conservative, population specific estimate of reliability. In this report, we demonstrate how interpretation of agreement can differ when using reliability estimates from either a standardization population (here test-retest reliability) or from the study population (here the intra-class correlation coefficient).

In order to provide such a population-specific estimate of reliability for our study, we calculated inter-rater reliability expressed as intra-class correlation coefficients (ICC). The intra-class correlation assesses the degree to which the measure used is able to differentiate between participants with diverging scores, indicated by two or more raters that reach similar conclusions using a particular tool (Liao et al., 2010; Kottner et al., 2011). Moreover, when considering extending the use of parental questionnaires to other caregivers, it is important to compare reliability between different rater groups. The ICC takes into account the variance of ratings for one child evaluated by two raters as well as the variance across the complete group of children. It can thus serve to compare the reliability of ratings between two groups of raters and to estimate the instrument's reliability in a concrete study. This study is the first to report inter-rater reliability assessed by intra-class correlations (ICCs) for the German vocabulary checklist ELAN (Bockmann and Kiese-Himmel, 2006).

In order to assess rater agreement, we first calculated two reliable change indexes (RCIs), one on the basis of the ELAN-manual's test-retest reliability, the second considering the ICC for our study population. Note that even though both reliability measures can be used to calculate the RCI, they are not equivalent in terms of accuracy and strictness. Test-retest correlations represent a very accurate estimate of the instrument's reliability (regarding a construct stable over time), inter-rater reliability rather reflects the rating process' accuracy. The proportion of (reliable) agreement was assessed using both reliability estimates in order to demonstrate how the choice of reliability measure impacts the evaluation and interpretation of rater agreement. In addition to the proportion of absolute agreement, information about the magnitude of (reliable) differences and about possible systematic direction of differences is also relevant for the comprehensive assessment of rater-agreement. Thus, three aspects of agreement are considered in this report: percentages of ratings that differ reliably, if applicable, the extent to which they differ, and the direction of the difference (i.e., a systematic response tendency of either group of raters compared to the other). In the analyses presented here we also relate magnitude of differences to those factors that can influence the likelihood of diverging ratings in our sample: gender of the evaluated child, bilingual vs. monolingual family environment and rater subgroup.

As shown above, Pearson correlations are the most commonly used statistic when inter-rater reliability in the domain of expressive vocabulary is assessed (e.g., Bishop and Baird, 2001; Janus, 2001; Norbury et al., 2004; Bishop et al., 2006; Massa et al., 2008; Gudmundsson and Gretarsson, 2009) and this tendency extends to other domains, such as language impairments (e.g., Boynton Hauerwas and Addison Stone, 2000), or learning disabilities (e.g., Van Noord and Prevatt, 2002). As argued above, linear correlations do not give information on ratings' agreement. However, they provide useful information on the relation between two variables, here vocabulary estimates of two caregivers for the same child. In the specific case of using correlation coefficients as an indirect measure of rating consistency, linear associations can be expected, thus Pearson correlations are an appropriate statistical approach. It cannot and should not serve as a sole measure of inter-rater reliability, but it can be employed as an assessment of strength of (linear) association. Correlation coefficients have the additional advantage of enabling comparisons, useful for example when examining between-group differences regarding the strength of ratings' association. Since most other studies assessing inter-rater reliability of expressive vocabulary scores report correlation coefficients (only), this measure also enables us to relate the results of the pre-sent study to earlier research. Thus, we report correlations for each of the two rating subgroups (mother–father and parent–teacher rating pairs), compare them and calculate the correlation of ratings across both subgroups, too.

In order to give one realistic, purposeful example of the research strategy outlined above, we employed the ELAN vocabulary scale (Bockmann and Kiese-Himmel, 2006), a German parental questionnaire, developed for screening purposes with regard to children's early expressive vocabulary. This instrument is comprised of a checklist including a total of 250 individual words: The rater decides for each item on the list whether or not the child actively uses it. General questions regarding demographic background and child development supplement the vocabulary information. Children experiencing regular daycare were evaluated by a daycare teacher and a parent, children cared for exclusively in their families were evaluated by both parents. Here, we provide a first analysis of the usability of the ELAN with daycare teachers and illustrate the necessity to evaluate rating scales on more than one dimension of rating consistency.

In summary, this report has two main goals: to provide a methodological tutorial for assessing inter-rater reliability, agreement and linear correlation of rating pairs, and to evaluate whether the German parent questionnaire ELAN (Bockmann and Kiese-Himmel, 2006) can be reliably employed also with daycare teachers when assessing early expressive vocabulary development. We compared mother–father and parent–teacher ratings with regard to agreement, correlation as well as reliability of ratings. We also explored which child and rater related factors influence rater agreement and reliability. In a relatively homogeneous group of mostly middle class families and high quality daycare environments, we expected high agreement and linear correlation of ratings.

## 2. Methods

### 2.1. Ethics statement

Parents, teachers and the heads of the child care centers participating in this study gave written informed consent according to the principles of the Declaration of Helsinki. Special care was taken to ensure that all participants understood that their participation is voluntary and could be ended at any time without causing them any disadvantages. The research reported here was conducted in Germany (country of residence of all authors) and met the Ethic Guidelines of the German Psychological Association and the German Psychological Professional Organization (Ethische Richtlinien der Deutschen Gesellschaft für Psychologie e.V. und des Berufsverbands Deutscher Psychologinnen und Psychologen e.V., see http://www.bdp-verband.org/bdp/verband/ethik.shtml), an approved German adaption of the “Ethical Principles of Psychologists and Code of Conduct” (American Psychological Association and Others, 2002).

### 2.2. Data collection, research instruments, exclusion criteria, and subgroups

Participating families and daycare centers were recruited from the German cities Konstanz and Radolfzell, as well as their surroundings. For each participating child, two caregivers assessed the number of spoken words on the basis of the German lexical checklist for parents ELAN (Bockmann and Kiese-Himmel, 2006). These two independent vocabulary ratings were provided within a period of 3 days before or after the child's second birthday. The data collection sessions with each of the two caregivers took place within a maximum of 6 days; more than 84% were completed within 48 h from each other. Data was collected by trained researchers from the University of Konstanz and was obtained for 59 two-year-old. The data of six children had to be excluded from further analyses due to the following reasons:

1. More than five missing answers to items of the vocabulary checklist (2). Respondents had to indicate, whether a child spoke a certain word by crossing either a “yes”- or a “no”-field, if no indication was provided, items were counted as “missing.”
2. Preterm birth (1).
3. State of transition between parental- and non-parental-care (1).
4. Vocabulary score too low to obtain a *T*-value (1).
5. Vocabulary information provided the maternal grandmother, instead of the father, as he did not speak any German (1).

Two independent vocabulary ratings for a total of 53 two-year-old children were included in the analyses. For those children (*n* = 34), who had experienced daily (Monday through Friday) non-parental care for at least 6 months, the two vocabulary ratings were provided by the daycare teacher responsible for each child in the daycare center and by one or two parents: either by the mother (27), or by the father (4), or by the mother and the father together (3). In this last case the two parents filled out one questionnaire actively communicating between each other about the answers and provided one single rating. We refer to the vocabulary rating pairs provided for these 34 children experiencing regular non-parental daycare as the “parent–teacher ratings.”

For those children (*n* = 19) who at the age of 2 years were cared for at home by their parents, the mother and the father each provided separate vocabulary ratings for their child. Data acquisition usually occurred at the same time, but special care was taken to ensure that the parents did not influence each other's responses. Children were also included in this group if they experienced some form of irregular non-parental care (e.g., playgroups or babysitters) up to a maximum of 12 h and up to three times per week. We refer to the vocabulary rating pairs provided by the mother and the father of the children experiencing parental care as the “parental” or “mother–father ratings.”

For all children vocabulary information was supplemented by demographic information provided by one parent (for a summary see Table 1). For children experiencing regular daycare additional information was provided by the daycare teacher concerning the duration and the quality of care (as indicated by the amount of time spent in direct proximity of the evaluated child, group size, teacher-to-child ratio, and educational background of the daycare teachers).

**Table 1:** **Demographic characteristics of the study population**.

	**Study population *N* (%)**	**Parent–teacher rating subgroup *n* (%)**	**Parental rating subgroup *n* (%)**	**Group Comparison**
Total number of children	53	34	19	
Female	29 (54.7)	21 (63.6)	8 (42.1)	n.s.
First born^a^	37 (69.8)	23 (67.6)	14 (73.7)	n.s.
Bilingual	12 (22.6)	10 (29.4)	2 (10.5)	n.s.
Two-parent household	45 (84.9)	26 (76.5)	19 (100)	*p* = 0.040
Highest sec. education: mothers	42 (79.2)	26 (76.5)	16 (84.2)	n.s.
Highest sec. education: fathers	41 (77.4)	27 (79.4)	14 (73.7)	n.s.
Mother employed	33 (62.7)	30 (88.2)	3 (15.8)	*p* < 0.001
Father employed	53 (100)	34 (100)	19 (100)	n.s.

a Including two pairs of first-born twins, all four children were counted as first born.

Notes: Percentages in brackets are group-based (column-wise). Group comparisons refer to Pearson's χ^2^-tests if expected values in all cells were above 4, otherwise, Fisher's Exact tests were employed.

Parental education level was defined as the highest school degree obtained. The category reported by the vast majority of the parents was the German university entrance certificate (Abitur) or a foreign equivalent and thus the highest possible secondary education degree in Germany (see Table 1). In addition, all parents had received further professional training and/or completed a high education degree. At the time of testing, mothers were either employed (33), on parental leave (18) or pursued a university degree (2). All fathers were employed.

All 53 two-year-old children spoke and listened to German on a daily basis, 41 of them were raised in monolingual German family environments (subsequently referred to as “monolingual” children). In contrast, 12 children had regular contact with a second language. One of those children was raised in a trilingual environment (the parents spoke two different languages other than German). Yet, we will refer to the complete group of 12 children as “bilingual.” All bilingual children actively spoke a second language in addition to German according to their parents.

A total of 24 daycare teachers participated in this study; four of them were the primary responsible teacher for more than one participating child and thus provided more than one evaluation. All of the participating teachers were female German native speakers. All but one daycare teacher had completed a vocational degree in early child-care, one teacher held a degree in nursing. All daycare teachers reported regular participation in continuing education courses. The group size in the daycare centers varied between 9 and 20 children, the majority (22 out of 34) were cared for in a group with up to 10 children and at least two daycare teachers present at all times. Weekly daycare reported by the parents varied between the categories “11–20 h” (*n* = 5) and “more than 20 h” (*n* = 28, one missing value).

The teachers participating in the study were always the ones who were primarily responsible for the evaluated children since their daycare enrollment. The daycare teachers provided information on the percentage of time spent in direct proximity, i.e., hearing and seeing the evaluated child. The teachers of 28 out of 34 children (82.35%) reported direct contact more than 60% of the time the evaluated child spent in daycare. The teachers of four children (11.76%) were in direct contact for 40–60% of time and only one child (2.94%) was reported to be in direct proximity to the evaluating teacher for 20–40% of daycare time; for one child, this data was missing.

### 2.3. Analyses

**Figure 1:**
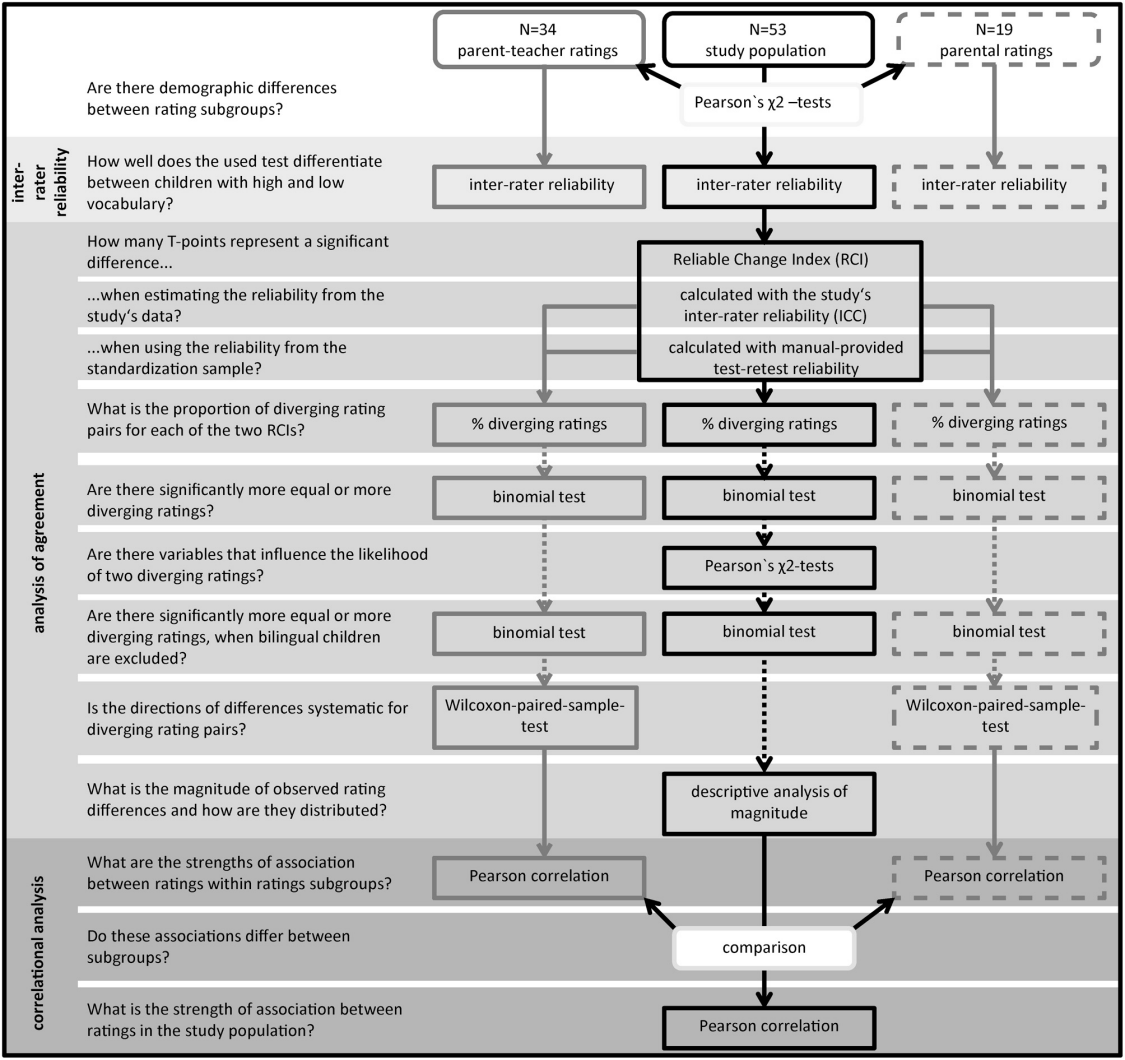
**Analysis procedure**. A total of 53 rating pairs was included in the analysis and divided in two rating subgroups (represented by round boxes in the upper line). On the left side of the figure the purpose of the applied statistical analysis is provided framed as research questions. The next column shows the analyses conducted within the parent–teacher rating subgroup (*n* = 34), in the right column the respective analyses for the mother–father rating subgroup (*n* = 19) are shown. The column in the middle lists tests conducted for the whole study population, as well as between group comparisons. Dotted arrows mark analyses conducted for the differing ratings identified using the manual's test-retest reliability (no reliably diverging ratings were identified if using the ICC for calculating the critical difference between ratings).

First, demographic differences between the two subgroups were assessed. Then inter-rater reliability, agreement and correlations within and across the two different rating subgroups were analyzed. The analysis procedure and the corresponding research questions are summarized in Figure 1.

Systematic demographic differences between the two rating subgroups were assessed regarding the following variables: educational level and occupational status of the parents, family status (one-parent- or two-parent-family), gender distribution, number of siblings, birth order, and number of bilingual children. If expected values in all cells were above 4, we used Pearson's χ^2^-tests, otherwise, Fisher's exact tests were employed.

Raw-vocabulary-scores were transformed into corresponding *T*-values according to the transformation table provided by the authors of the ELAN-questionnaire. All analyses were based on these standardized *T*-values.

We calculated inter-rater reliability for the mother–father as well as the parent–teacher rating subgroups and across the study population. We calculated the intra-class correlation coefficient as a measure of inter-rater reliability reflecting the accuracy of the rating process using the formula proposed by Bortz and Döring (2006), see also Shrout and Fleiss (1979):

(1) $$r_{ICC}=\left(\sigma_{bt}^{2}-\sigma_{in}^{2}\right)/\left(\sigma_{bt}^{2}+(k-1)*\sigma_{in}^{2}\right)$$

with σ^2^_*bt*_ being the variance of ratings between children, σ^2^_*in*_ being the variance within the children and *k* the number of raters. Confidence intervals for all ICCs were calculated in order to assess whether they differed from each other.

This analysis adds information regarding inter-rater reliability of the ELAN-questionnaire, and also serves as a basis for one out of two calculations of the reliable change index (RCI) considering the characteristics of the concrete study sample.

In order to determine, whether two ELAN ratings a child received differed statistically from one another, the RCI was calculated using the classical approach (Jacobson and Truax, 1991; Zahra and Hedge, 2010) as recommended e.g., in Maassen (2000), see also Maassen (2004) for a discussion about which exact formula should be used in which case.

(2) $$RCI=\left(x_{2}-x_{1}\right)/S_{diff}$$

with *x*_1_/*x*_2_ = compared scores and $S_{diff}=\sqrt{SEM^{2}}$. The latter gives the standard error of difference between two test scores and thus describes the spread of distribution of differences in case no differences actually occurred. *SEM* was calculated as $SEM=s_{1}\sqrt{1-r_{xx}}$, with *s*_1_ = *SD* and *r*_*xx*_ = reliability of measure.

RCI values are standardized *z*-values, therefore an RCI ≥ 1.96 indicates a difference at a significance level of α = 0.05. As all scores were transformed into standardized *T*-values, a *SD* of 10 was utilized.

For *r*_*xx*_ we used two different measures of reliability: (1) the *r*_*ICC*_ obtained across our study population and (2) the test-retest reliability provided in the ELAN-manual (Bockmann and Kiese-Himmel, 2006), a value originating from a larger and representative population and rather reflects the ELAN's and not our sample's characteristics. The use of external sources of reliability measures, as employed in the second RCI-calculation, has been recommended e.g., by Maassen (2004) and can be thought of as the most conservative means of estimating the RCI.

The RCI formula can be rearranged to determine the exact value from which onwards two *T*-values of the ELAN-questionnaire differ significantly:

(3) $$Diffx_{1}-x_{2}=1.96*\sqrt{2}\text{​}\left(s_{1}\sqrt{{\left(1-r_{xx}\right)}^{2}}\right)$$

Whether ratings differed significantly from each other was assessed within as well as between rating subgroups, proportions of diverging to equal ratings were calculated. If applicable, exact binomial tests were used to evaluate whether significantly more diverging than non-diverging ratings existed in each of the subgroups or across subgroups.

Pearson's χ^2^-tests were employed to determine whether the probability that a child received two diverging ratings differed for rater subgroups (mother–father vs. parent–teacher-ratings), for boys and girls as well as for mono- vs. bilingual two-year-old. We tested whether the differences' direction within each of the subgroups was systematic using Wilcoxon paired-sample tests.

We compared mean ratings for each of the different raters, i.e., parents and teachers for the 34 children experiencing daycare and for mothers and fathers for the 19 children in parental care using *t*-tests. In addition, the magnitude of individual differences was assessed descriptively. We displayed the distribution of differences with regard to the standard deviation of the *T*-distribution using a scatter plot (see Figure 3). Considering only children who received significantly diverging ratings, we also explored the magnitude of those differences by looking at the deviation between ratings of a pair using a graphical approach: a Bland-Altman plot (see Figure 4). A Bland-Altman plot, also known as Tukey mean-difference plot, illustrates dispersion of agreement by showing individual differences in *T*-values in relation to the mean difference. Therewith, magnitudes of differences in ratings can be categorized in relation to the standard deviation of differences (Bland and Altman, 2003).

To further assess the strength of linear relations between ratings, Pearson correlation coefficients were calculated for mother–father ratings and for parent–teacher ratings. In a next step, we assessed whether correlation coefficients of the two rating subgroups differed significantly from each other. For this statistical comparison, correlation coefficients were transformed into Fisher's *Z*-values, since means and standard deviations of correlation coefficients cannot be compared directly (see for example, Bortz and Döring, 2006). A Pearson correlation coefficient was also obtained for the whole study population, in order to assess the general strength of linear association between two different raters. To make this calculation possible, we combined teacher—with maternal ratings and parental with paternal ratings.

## 3. Results

### 3.1. Comparison of demographic characteristics between rating subgroups

There were no significant differences between rating subgroups (and thus between children experiencing early center based daycare and children cared for exclusively at home) regarding parental education (mothers and fathers), occupational status of the father, number of siblings, birth order, gender distribution and number of bilingual children, all *p* ≥ 0.05. The employment status of the mother differed significantly between subgroups (χ^2^(1, *N* = 53) = 27.226, *p* < 0.001), as did the number of children raised in two-parent-, as opposed to single-parent-households (χ^2^(1, *N* = 53) = 5.265, *p* = 0.040); see Table 1 for absolute numbers and percentages. This means, that children in the two rating subgroups did not differ regarding most demographic variables. Importantly, we did not find systematic differences in parental education, gender distribution and birth order. The observed divergences regarding family and employment status are explicable by the fact that children below the age of three could only enter center-based state-regulated daycare facilities in the cities of Konstanz and Radolfzell, if the parents (or in the case of a single-parent family the one parent) were employed, pursuing their education, or were currently on parental leave with a younger child.

### 3.2. Inter-rater reliability

**Figure 2:**
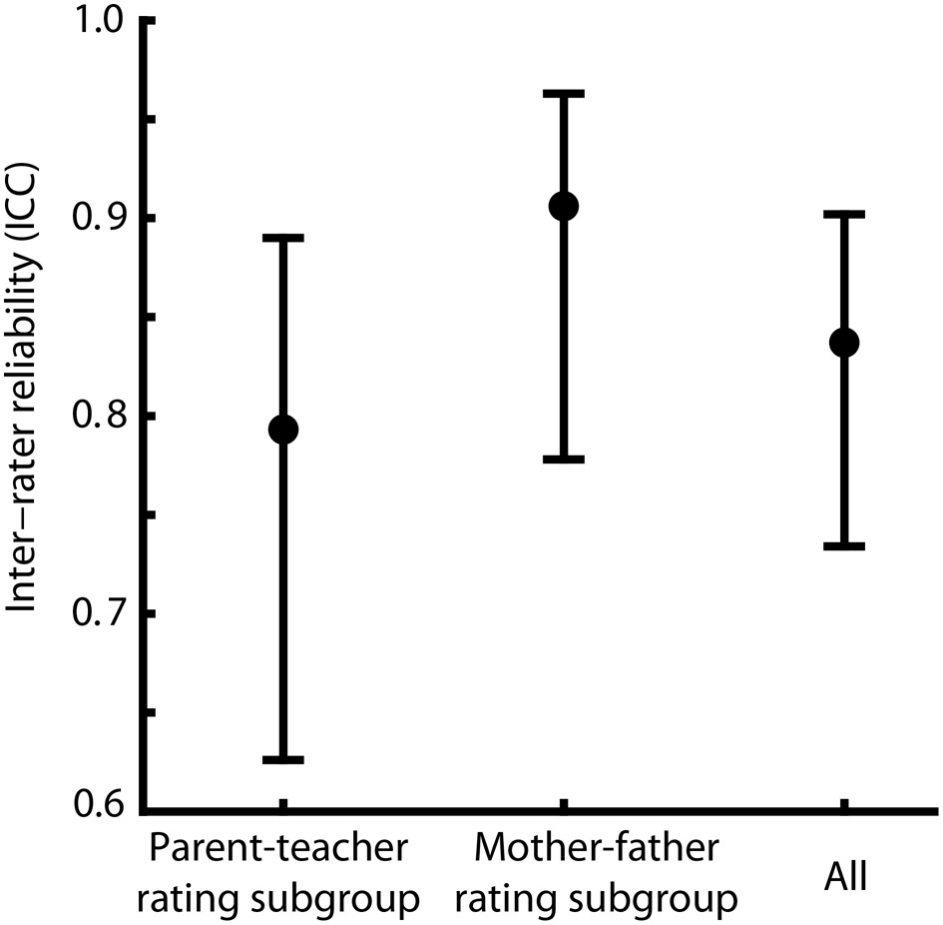
**Comparison of inter-rater reliability**. Intra-class correlation coefficients (ICCs, represented as dots) and corresponding confidence intervals at α = 0.05 (CIs, represented as error bars) for parent–teacher ratings, mother–father ratings and for all rating pairs across rater subgroups. Overlapping CIs indicate that the ICCs did not differ systematically from each other.

Inter-rater reliability was calculated within subgroups and across the study population as an estimate for the accuracy of the rating process. For the mother–father rating subgroup the intra-class correlation coefficient (ICC) was *r*_*ICC*_ = 0.906, for the parent–teacher-rating subgroup an ICC of *r*_*ICC*_ = 0.793 was found. Across the study population the calculation of the ICC resulted in a reliability of *r*_*ICC*_ = 0.837. The confidence intervals (α = 0.05) of reliabilities for the subgroups and for the study population are overlapping, indicating that they do not differ from each other (see Figure 2 for ICCs and the corresponding confidence intervals). Thus, we did not find evidence that the ability of the ELAN to differentiate between children with high and low vocabulary is lowered when instead of two parents a parent and a teacher provide evaluations.

### 3.3. Number, likelihood, and direction of rating differences

The Reliable Change Index (RCI) was used to calculate the least number of *T*-points necessary for two ELAN-scores to be significantly different from each other. We used two different estimates of reliability to demonstrate their impact on measures of agreement. First, the ICC calculated across the complete study population was employed as an estimate for the ELAN's reliability in this concrete study's population. As the ICC is calculated within and between subjects and not between specific rater groups, this is a valid approach for estimating overall reliability across both rating subgroups.

The critical difference when considering the ICC calculated across the study population The critical difference was $Diff_{T_{1}\;-\;T_{2}}=1.96*\sqrt{2}(10^{2}\sqrt{{\left(1-0.837\right)}^{2}})=11.199$. Since *T*-scores are calculated in integral numbers only, this result means that for the ELAN-questionnaire two ratings differ statistically at a significance level lower than α = 0.05, if the difference between them equals or is greater than 12 *T*-points.

When using the reliability provided in the ELAN-manual (Bockmann and Kiese-Himmel, 2006), and thus when employing a more conservative estimate of reliability, the RCI was considerably lower, $Diff_{T_{1}\;-\;T_{2}}=1.96*\sqrt{2}(10^{2}\sqrt{{\left(1-0.99\right)}^{2}})=2.772$, resulting in a critical difference of three *T*-points.

Measuring the reliable difference between ratings on the basis of the inter-rater reliability in our study resulted in 100% rating agreement. In contrast, when the RCI was calculated on the basis of the manuals' more conservative test-retest reliability, a substantial number of diverging ratings was found; absolute agreement was 43.4%. When this conservative estimate of the RCI was used, significantly higher numbers of equal or diverging ratings were not found, neither for a single rating subgroup, nor across the study population. (see Table 2 for the results of the relevant binomial tests). Thus, the probability of a child to receive a concordant rating did not differ from chance. When the study's own reliability was employed, the probability to receive concordant ratings was 100% and thus clearly above chance.

**Table 2:** **Proportions of diverging ratings for monolingual, bilingual, and all children in the sample**.

	**All children/monolingual children/bilingual children**			
	**Number of diverging ratings**	**% of diverging ratings**	**Sample size**	***p*-value**
Parent–teacher rating subgroup	21/12/9	61.8/50.0/90.0	34/24/10	1/0.230/0.021
Parental rating subgroup	9/7/2	47.4/41.2/100	19/17/2	1/0.629/0.500
Study population	30/19/11	56.6/46.3/91.7	53/41/12	0.410/0.755/0.006

Notes: To facilitate comparison, the numbers in the columns are provided for whole (sub-) sample (left side), monolingual children (middle) and bilingual children (right side).

In the parent–teacher rating subgroup 21 out of 34 children received diverging ratings; 9 out of 19 children received diverging ratings in the mother–father rating subgroup. Binomial tests (see Table 2 for details) clarified that these absolute differences were not statistically reliable within the limitations posed by the small sample size.

### 3.4. Factors influencing the likelihood and direction diverging ratings

The results reported in this section consider those rating pairs that were classified as reliably different using the more conservative RCI calculation on the basis of the test-retest reliability, which yield a considerable number of diverging ratings. We explored the potential influence of three different factors on the likelihood of receiving diverging ratings: rating subgroup (mother–father vs. teacher–parent), gender of the child and bilingualism of the child.

The likelihood to receive diverging ratings did not depend systematically on whether a child was evaluated by a teacher and a parent or by father and mother [χ^2^(1, *N* = 53) = 1.028, *p* = 0.391]. Being a boy or a girl also did not change the likelihood of receiving diverging ratings [χ^2^(1, *N* = 53) = 0.106, *p* = 0.786]. In contrast, monolingual and bilingual children differed significantly concerning the likelihood of receiving two different ratings [χ^2^(1, *N* = 53) = 7.764, *p* = 0.007]: Bilingual children (*n* = 12, 11 different ratings) were much more likely to receive diverging scores than monolingual children (*n* = 41, 19 different ratings).

Next, we assessed whether the likelihood to receive diverging ratings was above chance. We conducted these binomial tests separately for bilingual and monolingual children, as bilingual children were shown to receive more diverging ratings compared to monolingual children. As only 2 out of 19 bilingual children were rated by two parents (see Table 1), we also considered rating subgroups separately. As summarized in Table 2, the likelihood to receive diverging ratings exceeded chance for bilingual children only. However, conclusions about whether this is also true for bilingual children rated by two parents cannot be drawn on the basis of our data, as only two children fell in this category.

Wilcoxon paired-sample tests were used to uncover possible systematic direction tendencies for different groups of raters. None of the within subgroup comparisons (maternal- vs. paternal- and teacher- vs. parent-ratings) reached significance (all *p* ≥ 0.05). Thus, we did not find evidence for systematic direction of rating divergence, neither for bilingual, nor for monolingual children.

We therefore conclude that within the two different rating subgroups a similar proportion of diverging ratings occurred. Neither the gender of the child, nor whether the expressive vocabulary was evaluated by two parents or by a teacher and a parent, increased the probability of the children to receive two diverging ratings. The only factor that reliably increased this probability was bilingualism of the child. No systematic direction of differences was found.

### 3.5. Comparison of rating means and magnitude of differences

In a first step, we compared means of ratings for each rater group: mothers, fathers, parents and teachers. *T*-Tests did not reveal any significant differences (see Table 3).

**Table 3:** **Means and standard deviations of vocabulary ratings and comparisons of means**.

**Rater group (sample size) mean, standard deviation**	**Teacher**	**Mother**	**Father**
Parent (*n* = 34)	*t*_(66)_ = −0.29,	*t*_(51)_ = −1.67,	*t*_(51)_ = −1.00,
*M* = 49.26, *SD* = 6.94	*p* = 0.771	*p* = 0.101	*p* = 0.322
Teacher (n = 34)	–	*t*_(51)_ = −1.29,	*t*_(51)_ = −0.69,
*M* = 49.79, *SD* = 7.99		*p* = 0.203	*p* = 0.495
Mother (*n* = 19)	–	–	*t*_(36)_ = 0.52,
*M* = 52.68, *SD* = 7.53			*p* = 0.605
Father (*n* = 19)	–	–	–
*M* = 51.37, *SD* = 8.03			

**Figure 3:**
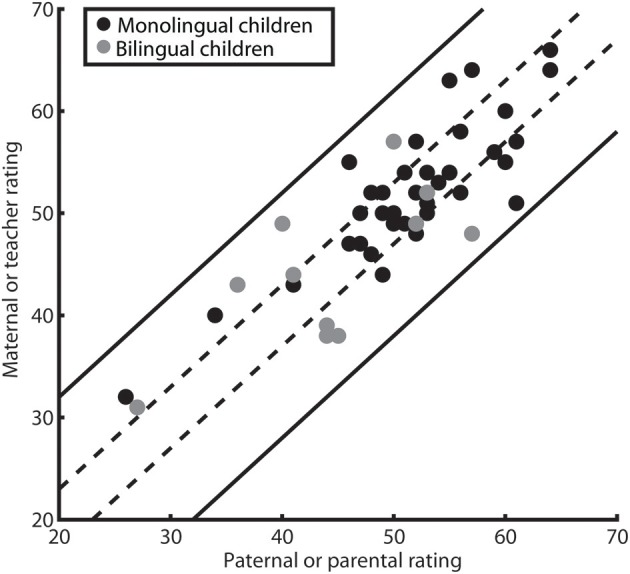
**Scatter-plot of children's ratings**. Every dot represents two ratings provided for a child. For the parent–teacher rating subgroup, parental ratings are on the *x*-axis, teacher ratings are on the *y*-axis, for the parental rating subgroup, paternal ratings are on the *x*-axis, maternal ratings are on the *y*-axis. Ratings for bilingual children are represented by gray, for monolingual children by black dots. Dashed lines enclose statistically identical ratings as calculated on the basis of the manual-provided test-retest reliability (less than 3 *T*-points difference; 23 out of 53 rating pairs). Straight lines enclose statistically identical ratings as calculated on the basis of the inter-rater reliability (ICC) in our study (less than 12 *T*-points difference).

**Figure 4:**
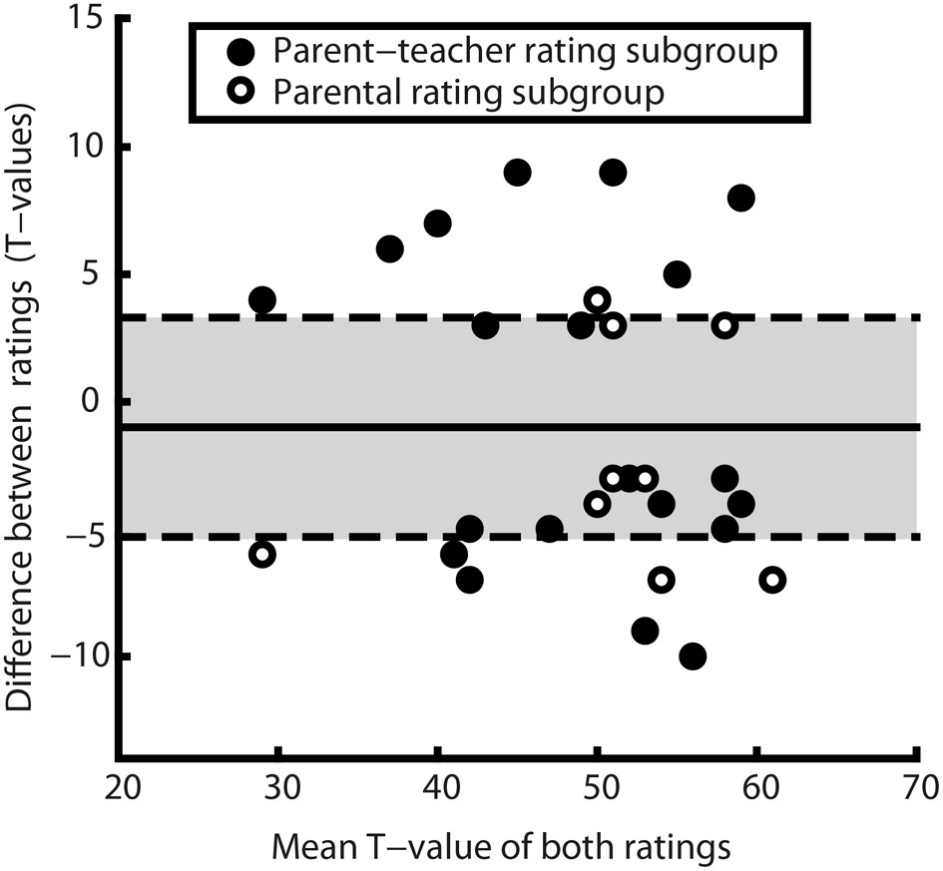
**Bland-Altman plot of *T*-values, corresponding to a Tukey mean-difference plot**. The solid line indicates the mean difference (*M* = −1), dashed lines mark mean difference ±1.96 SDs. Dots represent the 30 rating pairs diverging significant in the study population. Differing mother–father ratings are represented by empty, differing parent–teacher ratings by filled dots. Positive differences indicate a higher evaluation of the parent in the parent–teacher rating subgroup or a higher evaluation by the father in the parental rating subgroup (*M* = −1, *SD* = 5.7, min = −10, max = 9). Note that all but one difference lie within in the range of ± 10 *T*-points (1 SD on a *T*-scale) and that there is no indication for systematic over- or underrating.

Only when using the test-retest reliability provided in the manual of the ELAN, there was a substantial number of differing rating pairs (30 out of 53 or 56.6%). The magnitude of these differences was assessed descriptively using a scatter plot (see Figure 3) and a Bland-Altman plot (also known as Tukey mean-difference plot, see Figure 4). First, we displayed the rating of the individual children in a scatter plot and illustrated the two different areas of agreement: 43.4% of ratings diverged by less than three *T*-points and can thus be considered concordant within the limits of the more conservative RCI estimate, all 100% of the ratings lie within 11 *T*-points and thus within the limits of agreement based on a reliability estimate obtained with the present study's sample.

Another way of illustrating the magnitude of differences is to display the distribution of significant differences, where mean *T*-values are plotted against the absolute difference values as proposed by Bland and Altman (1986, 2003). This plot (see Figure 4) shows that 18 out of 30 observed differences (60%) are within 1 *SD* of differences (*SD* = 5.7). The limits of agreement in this study, as defined by Bland and Altman (2003), to contain 95% of the differences in similar populations are −12.2 to 10.2 *T*-points, a range that contains all of the observed differences in this study. Thus, the graphical approach toward assessing differences' magnitude mirrors the result of 100% rater agreement if considering ICC as the reliability in the calculation of reliable differences.

### 3.6. Correlations between ratings

So far we reported results regarding inter-rater reliability and the number of diverging ratings within and between subgroups using two different but equally legitimate reliability estimates. We also explored which factors might influence the likelihood of receiving two statistically diverging ratings and described the magnitude of observed differences. These analyses focused on inter-rater reliability and agreement, as well as related measures. In this last section we turn to Pearson correlations coefficients in order to explore the linear relation between ratings and their strength within and between rater subgroups.

**Figure 5:**
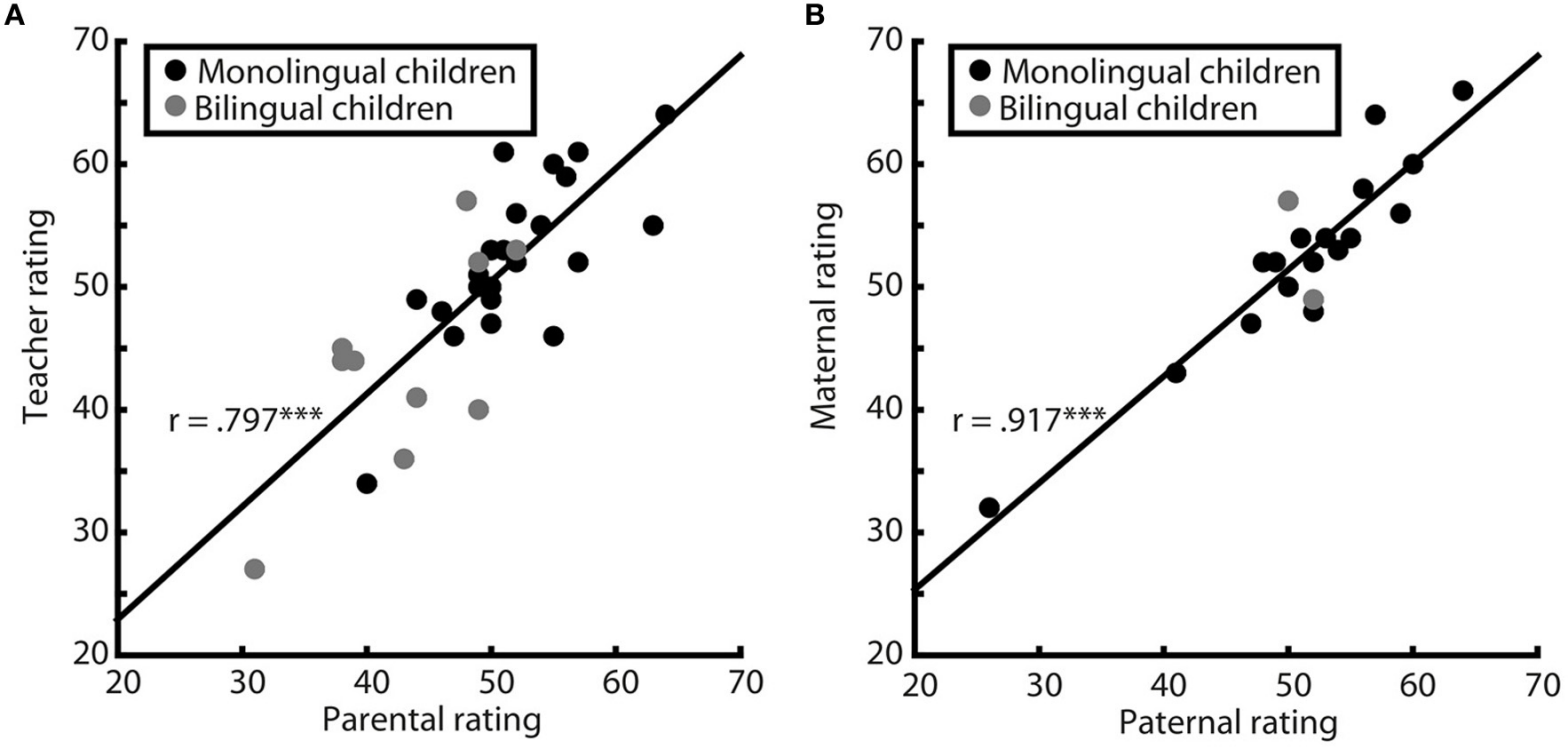
**Correlations of ratings**. Pearson correlations of parent–teacher ratings (**A**, *n* = 34) and of mother–father ratings (**B**, *n* = 19), both significant (both *p* ≤ 0.001) and with large effect sizes. Monolingual children are represented by black, bilingual by gray dots. The two correlations did not differ significantly from each other (*p* = 0.119). ^***^p < 0.001.

Teacher and parent ratings were highly correlated [*r* = 0.797, *p* < 0.001, 95% CI (0.503, 1.0), see Figure 5A] with large effect size of *R*^2^ = 0.636. For the mother–father rating subgroup correlation between maternal and paternal ratings was similarly high [*r* = 0.917, *p* < 0.001, 95% CI (0.698, 1.0), see Figure 5B], effect size of *R*^2^ = 0.842. The strength of relation between ratings did not differ systematically between the two rating subgroups (*p* = 0.119). For the whole study population (*n* = 53) Pearson correlation between ratings of two different caregivers was *r* = 0.841, *p* < 0.001, *R*^2^ = 0.707. In conclusion, with regard to correlation of ratings, strong associations were observed for ratings provided by mothers and fathers, as well as for those provided by teachers and parents and thus across our study sample.

## 4. Discussion

In this report a concrete data set is employed to demonstrate how a comprehensive evaluation of inter-rater reliability, inter-rater agreement (concordance), and linear correlation of ratings can be conducted and reported. On the grounds of this example we aim to disambiguate aspects of assessment that are frequently confused and thereby to contribute to increasing comparability of future rating analyses. By providing a tutorial, we hope to foster knowledge transfer to e.g., educational and therapeutic contexts, in which the methodological requirements for rating comparison are disregarded still too frequently, leading to misinterpretation of empirical data.

We analyzed two independent vocabulary ratings obtained for 53 German speaking children at the age of 2 years with the German vocabulary scale ELAN (Bockmann and Kiese-Himmel, 2006). On the example of assessing whether ELAN ratings can be reliably obtained from daycare teachers as well as from parents we show that rater agreement, linear correlation, and inter-rater reliability all have to be considered. Otherwise, an exhaustive conclusion about a rating scale's employability with different rater groups cannot be made. We also considered the factors gender and bilingualism of the evaluated child as potentially influencing the likelihood of rating agreement.

First, we assessed the inter-rater reliability within and across rating subgroups. The inter-rater reliability as expressed by intra-class correlation coefficients (ICC) measures the degree to which the instrument used is able to differentiate between participants indicated by two or more raters that reach similar conclusions (Liao et al., 2010; Kottner et al., 2011). Hence, the inter-rater reliability is a quality criterion of the assessment instrument and the accuracy of the rating process rather than one quantifying the agreement between raters. It can be regarded as an estimate for the instrument's reliability in a concrete study population. This is the first study to evaluate inter-rater reliability of the ELAN questionnaire. We report high inter-rater reliability for mother–father as well as for parent–teacher ratings and across the complete study population. No systematic differences between the subgroups of raters were found. This indicates that using the ELAN with daycare teachers does not lower its capability to differentiate between children with high and low vocabulary.

The term “agreement” describes the degree to which ratings are identical (see for example, de Vet et al., 2006; Shoukri, 2010; Kottner et al., 2011). Many studies supposedly evaluating agreement of expressive vocabulary ratings rely (only) on measures of strength of relations such as linear correlations (e.g., Bishop and Baird, 2001; Janus, 2001; Van Noord and Prevatt, 2002; Bishop et al., 2006; Massa et al., 2008; Gudmundsson and Gretarsson, 2009). In some studies the raw scores are used as reference values and critical differences are disregarded (e.g., Marchman and Martinez-Sussmann, 2002; McLeod and Harrison, 2009). However, absolute differences between raw scores or percentiles do not contain information about their statistical relevance. We demonstrate the use of the Reliable Change Index (RCI) to establish statistically meaningful divergences between rating pairs. We obtained two different RCIs on the basis of two reliability measures: the test-retest reliability provided in the ELAN's manual (Bockmann and Kiese-Himmel, 2006) and the inter-rater reliability (expressed as ICC) derived from our sample. This dual approach was chosen to demonstrate the impact of more or less conservative, but similarly applicable reliability estimates, on measures of rating agreement. We determined that, if considering the reliability provided in the ELAN-manual, ratings differ reliably if the absolute difference between them amounts to three or more *T*-points. With regard to the reliability of our study, however, this difference necessary to establish reliable divergence between two ratings is considerably larger, i.e., 12 *T*-points or more.

For both critical values we determined absolute agreement (e.g., Liao et al., 2010) as the proportion of statistically non-different ratings. Absolute agreement was 100% if considering the RCI calculated on the basis of the ICC for our sample. In contrast, absolute agreement was 43.4% if the manual's test-retest reliability was used to estimate the critical difference. With this more conservative measure of absolute agreement, the probability to receive a concordant rating did not differ from chance. This probability did not differ statistically for the two rating subgroups (parent–teacher and mother–father ratings) and thus across the study population, regardless of the chosen RCI calculation. These results support the assumption that parents and daycare teachers in this case were similarly competent raters with regard to early expressive vocabulary of the children. Nonetheless, the RCIs obtained with different reliability estimates differ substantially with regard to the specific estimates of absolute agreement. The profoundly diverging amounts of absolute agreement obtained by using either inter-rater reliability within a relatively small sample or the instrument's test-retest reliability obtained with a large and more representative sample highlights the need for caution when calculating reliable differences.

Absolute agreement of 100% can undoubtedly be considered high. Whether 43.4% proportion of absolute agreement is high or low needs to be evaluated in comparison to previous reports using similar instruments and methods of analyses. In the domain of expressive vocabulary, however, we scarcely find empirical studies reporting the proportion of absolute agreement between raters. If they do, they consider agreement on the level of individual items (here words) and not on the level of the overall rating a child receives (de Houwer et al., 2005; Vagh et al., 2009). In other domains, such as attention deficit or behavior problems, percentages of absolute agreement as proportion of concordant rating pairs are reported more often and provide more comparable results (e.g., Grietens et al., 2004; Wolraich et al., 2004; Brown et al., 2006). In those studies, agreement is considered high at and above 80% absolutely agreeing rating pairs; proportions of absolute agreement below 40% are considered low. However, one should take into account that these studies usually evaluate inter-rater agreement of instruments with far fewer items than the present study in which raters had to decide on 250 individual words. When comparing the results of our study and those of studies in other domains it has to be considered that increasing the number of items composing a rating reduces the likelihood of two identical scores. The difficulty to find reliable and comparable data on rater agreement in the otherwise well-examined domain of early expressive vocabulary assessment highlights both the widespread inconsistency of reporting practices and the need to measure absolute agreement in a comparable way, as e.g., presented here.

In order to evaluate inter-rater agreement in more detail, the proportion of absolute agreement needs to be considered in light of magnitude and direction of the observed differences. These two aspects provide relevant information on how close diverging ratings tend to be and whether systematically higher or lower ratings emerge for one subgroup of raters or rated persons in comparison to another. The magnitude of difference is an important aspect of agreement evaluations, since the proportions of statistically equal ratings only reflect perfect concordance. Such perfect concordance may, however, not always be relevant, e.g., by clinical means. In order to assess the magnitude of difference between raters, we employed a descriptive approach considering the distribution and the magnitude of score differences. As reliably different ratings were only observed when calculations were based on the test-retest reliability of the ELAN, we used these results to assess magnitude and direction of differences. Overall, the differences observed were small: most of them (60%) within 1 *SD*, all of them within 1.96 *SDs* of the differences' mean. Thus, the occurring differences were in an acceptable range for a screening tool, since they did not exceed one standard deviation of the norm scale used. This finding puts into perspective the relatively low proportion of absolute agreement measured on the groups of the tools test-retest reliability (43.4%) and highlights the importance of not only considering significance but also magnitude of differences. Interestingly, it is also in line with the 100% absolute agreement resulting from calculations employing this study's rather than the standardized reliability of the instrument used.

The analysis of differences' direction is intended to uncover systematic rating tendencies by a group of raters or for a group of rated persons. Some validity studies show a tendency of raters, specifically of mothers, to estimate children's language developmental status higher than the results obtained via objective testing of the child's language abilities (Deimann et al., 2005; Koch et al., 2011; Rennen-Allhoff, 2012). Whether these effects reflect an overrating of the abilities of the children by their mothers, or the fact that objective results acquired specifically for young children might underestimate the actual ability of a child, remains uncertain. In the present study we did not assess validity and thus did not compare the acquired ratings to objective data. This also means that our assessments cannot reveal lenience or harshness of ratings. Instead, comparisons were conducted between raters, i.e., between mother and father, as well as between teacher and parent. We did not find any systematic direction of differences under these circumstances: No one party of either rating pair rated children's vocabulary systematically higher or lower than the other.

As explained above, only with the more conservative approach to calculate the RCI did we find a substantial amount of diverging ratings. We looked at the factors possibly influencing the likelihood of receiving diverging ratings. Neither gender of the child, nor whether it was evaluated by two parents or by a parent and a teacher, influenced this likelihood systematically. Bilingualism of the evaluated child was the only examined factor which increased the likelihood of a child to receive diverging scores. It is possible that diverging ratings for the small group of bilingual children reflected systematic differences of vocabulary used in the two different settings: monolingual German daycare and bilingual family environments. Larger groups and more systematic variability of the bilingual environment characteristics are necessary to determine whether bilingualism has a systematic effect on rater agreement, as suggested by this report and, if yes, where this effect stems from.

In order to further explore the linear relation between ratings, we calculated Pearson correlation coefficients. As mentioned above, many researchers employ correlation coefficients as an indicator of agreement (e.g., Bishop and Baird, 2001; Janus, 2001; Van Noord and Prevatt, 2002; Norbury et al., 2004; Bishop et al., 2006; Massa et al., 2008; Gudmundsson and Gretarsson, 2009), disregarding the fact that correlation measures the strength of the relation between two variables or ratings, but does not in itself provide information on the extent of agreement between them (for a methodological background see for example, Liao et al., 2010; Kottner et al., 2011). However, Pearson correlation coefficients are useful when quantifying the strength of linear association between variables. They can also be compared to assess differences between rater groups concerning these relations. In the context of vocabulary assessment, they allow us to relate the present results to previous findings. We found high correlation coefficients (*r* = 0.841) across the study population and within each of the two rating subgroups (parent–teacher ratings *r* = 0.797, mother–father ratings *r* = 0.917). These correlations are higher than those found in comparable studies which are mostly moderate with correlation coefficients ranging from *r* = 0.30 to *r* = 0.60 Bishop and (Baird, 2001; Janus, 2001; Norbury et al., 2004; Bishop et al., 2006; Massa et al., 2008; Gudmundsson and Gretarsson, 2009; Koch et al., 2011). Possible explanations can be found in our population characteristics, specifically in the homogeneity of the children's family and educational backgrounds, as well as the high professional qualification of the teachers in the participating state regulated daycare facilities. The high correlations could also be seen as indication that the employed questionnaire was easy to understand and unambiguous for most of the raters. What is more, we did not find differences in correlation coefficients when comparing rater subgroups. These results provide evidence that two parental ratings were not more strongly associated with each other than a parent with a teacher rating and that in general the two ratings of the expressive vocabulary of a child obtained with the ELAN-questionnaire (Bockmann and Kiese-Himmel, 2006) were strongly associated with each other.

Taking together the results on agreement and those on linear correlations, we conclude that both measures are important to report. We demonstrate that high correlations of ratings do not necessarily indicate high agreement of ratings (when a conservative reliability estimate is used). The present study is an example of low to moderate agreement of ratings combined with relatively small magnitude of differences, unsystematic direction of differences and very high linear correlations between ratings within and between rating subgroups. In our study it would have thus been very misleading to only consider correlations as a measure of agreement (which they are not).

In summary, this study provides a comprehensive evaluation of agreement within and between two rater groups with regard to a German expressive vocabulary checklist for parents (ELAN, Bockmann and Kiese-Himmel, 2006). Inter-rater reliability of the ELAN-questionnaire, assessed here for the first time, proved to be high across rater groups. Within the limits of population size and its homogeneity, our results indicate that the ELAN-questionnaire, originally standardized for parents, can also be used reliably with qualified daycare teachers who have sufficient amount of experience with a child. We did not find any indication for systematically lower agreement of parent–teacher ratings compared to mother–father ratings. Also, teachers compared to parents as well as mothers compared to fathers did not provide systematically higher or lower ratings. The magnitude of absolute agreement profoundly depended on the reliability estimate used to calculate a statistically meaningful difference between ratings. The magnitude of rating differences was small and the strength of association between vocabulary ratings was high. These findings highlight that rater agreement has to be assessed in addition to correlative measures while not only taking significance but also magnitude of differences into account.

The employed and discussed analytical approach serves as one example for evaluation of ratings and rating instruments applicable to a variety of developmental and behavioral characteristics. It allows the assessment and documentation of differences and similarities between rater and rated subgroups using a combination of different statistical analyses. If future reports succeed in disambiguating the terms agreement, reliability and liner correlation and if the statistical approaches necessary to tackle each aspect are used appropriately, higher comparability of research results and thus improved transparency will be achieved.

## Funding

Funding for this study was provided by the Zukunftskolleg of the University of Konstanz.

### Conflict of interest statement

The authors declare that the research was conducted in the absence of any commercial or financial relationships that could be construed as a potential conflict of interest.
